# Histopathological features of gastric map-like redness under image-enhanced endoscopy

**DOI:** 10.3389/fmed.2026.1833606

**Published:** 2026-07-06

**Authors:** Yixiang You, Jin Zheng, Wulian Lin, Shentong Yu, Chao Gao, Yunmeng Zheng, Guanpo Zhang, Wen Wang

**Affiliations:** 1 Department of Gastroenterology, Fuzong Clinical Medical College of Fujian Medical University, Fuzhou, China; 2 Department of Gastroenterology, 900th Hospital of PLA Joint Logistic Support Force, Fuzhou, China; 3 Department of Gastroenterology, Zhangzhou Affiliated Hospital of Fujian Medical University, Zhangzhou, China; 4 Department of Pathology, 900th Hospital of PLA Joint Logistic Support Force, Fuzhou, China

**Keywords:** gastric cancer, gastric intestinal metaplasia, Helicobacter pylori, image-enhanced endoscopy, map-like redness

## Abstract

**Background and study aims:**

Map-like redness (MLR) is a characteristic feature of gastritis after *Helicobacter pylori* (*H. pylori*) eradication and independent risk factor of gastric cancer after *H. pylori* eradication. The present study investigated the endoscopic manifestations and histopathological features of MLR to further investigate the relationship between MLR and gastric cancer after *Helicobacter pylori* eradication.

**Patients and methods:**

We enrolled 56 consecutive MLR patients between January 2023 and July 2023. Endoscopic signs and histopathological characteristics were compared among the reddish area, transition zone, and background mucosa (areas R, T, and B, respectively).

**Results:**

The histopathological analysis showed significantly greater atrophy, gastric intestinal metaplasia (GIM), and dysplasia in area R compared to other areas (*p* < 0.05). Incomplete GIM predominated in area R, while complete GIM predominated in areas T and B. Area R had a higher microvascular density compared to areas T and B. Intervening parts width for area T was greater than that for area B, which was greater than that for area R (*p* < 0.05). Crypt opening sizes in area R were greater than those in area T, which were greater than those in area B (*p* < 0.05).

**Conclusion:**

Gastric MLR, particularly the central reddish area, was associated with localized high-risk mucosal changes, including advanced atrophy, GIM, incomplete GIM, increased microvessel density, and dysplasia in some cases. MLR may serve as an endoscopic marker of high-risk mucosal changes after successful *H. pylori* eradication.

## 1 Introduction

Gastric cancer is the fifth most common cancer and fourth leading cause of cancer-related deaths worldwide. According to global cancer burden data from 2020, gastric cancer ranks third in both incidence and mortality in China (1). With widespread *H. pylori* eradication, the incidence of gastric cancer has decreased (2, 3). However, some atrophic gastritis patients still progress to gastric cancer despite successful *H. pylori* eradication (4, 5). Take et al. (6) reported an annual incidence of 0.3% for gastric cancer after *H. pylori* eradication, indicating a persistent risk. Published data indicate that the incidence of gastric cancer after *H. pylori* eradication can reach up to 7.2% (7), with the risk persisting for approximately 10–14.5 years (8). Previous clinical studies (9, 10) have identified MLR as an independent risk factor for gastric cancer following eradication, with a 1.75–3.62 times higher risk in patients exhibiting MLR compared to controls. In recent years, increased attention has been paid to gastric cancer cases detected after eradication, including those occurring after eradication and those occurring before but detected after *H. pylori* eradication, with a particular focus on cases detected more than a year after *H. pylori* eradication (11). Several studies have identified MLR as an independent risk factor for gastric cancer after *H. pylori* eradication (9, 12–14). The manifestations of MLR [also called “color reversal” phenomenon or mottled patchy erythema (MPE)] result from *H. pylori*-associated gastritis with improvements following *H. pylori* elimination through the use of antibiotics or severe gastric mucosal atrophy. The endoscopic appearance of diffuse redness disappears and the color contrast between non-atrophic and atrophic gastritis increases, resulting in color reversal with more pronounced redness in some mucosal areas, termed as MLR in the Kyoto classification of gastritis (11, 15). Nagata et al. (12) reported GIM as the pathological basis of MLR, serving as a specific indicator for gastritis after eradication with a sensitivity of 72.7% and a specificity of 84.1%. However, the specific endoscopic manifestations and pathological characteristics remain unclear, prompting the need for this study, to further investigate the relationship between MLR and gastric cancer after *Helicobacter pylori* eradication.

## 2 Patients and methods

### 2.1 Patients

This prospective study was conducted at the 900th Hospital of PLA between January 2023 and July 2023, and included patients diagnosed with MLR during electronic gastroduodenoscopy. The study was approved by the Medical Science Ethics Committee of the hospital (approval No. 2023–029). Patients and their families were informed about the potential risks and benefits of participation, and signed informed consent forms.

The inclusion criteria included patients with a documented history of *H. pylori* infection, confirmed by a positive carbon-13 breath test, rapid urease test, or histopathological *H. pylori* test, and successful eradication for more than 1 year, confirmed by negative carbon-13 breath test, rapid urease test, and histopathological *H. pylori* test results. MLR was operationally defined as multiple well-demarcated, map-like or mottled reddish lesions observed under white-light imaging (WLI) after successful *H. pylori* eradication. The lesions were flat or slightly depressed, showed a relatively uniform reddish color compared with the surrounding mucosa, and were distinguishable from diffuse hyperemia or non-specific patchy erythema by their clear borders and map-like configuration. Exclusion criteria were individuals with advanced gastric cancer, a history of subtotal gastrectomy, recent upper gastrointestinal bleeding or coagulation dysfunction, and conditions that could affect upper gastrointestinal endoscopy, biopsy, or endoscopic resection.

### 2.2 Image enhanced endoscopy and biopsy sampling

Senior physicians, having an experience of over 1,000 image-enhanced gastroscopies using linked color imaging (LCI) and blue laser imaging (BLI), initially conducted a thorough examination of the entire stomach using the WLI mode of the FUJIFILM system, and categorized different signs of MLR into two areas: the central slightly depressed reddish area (area R), and the surrounding slightly rough microprotruded transition zone (area T). Additionally, the peripheral flat, smooth orange-red or red-white background mucosa was termed the background mucosa area (area B). Subsequently, LCI, BLI and magnifying blue laser imaging (M-BLI) were used to meticulously examine the specific and detailed features of MLR and the surrounding mucosa, respectively. Meanwhile, endoscopists recorded the microsurface (MS; oval, tubular, villous, or gyral) and microvessel (MV; regular, irregular, morphology: mesh- or coil-like) patterns. Subsequently, biopsy samples (two biopsies per point) were obtained for each area, and in selected cases, endoscopic submucosal dissection (ESD) or endoscopic mucosal resection (EMR) was performed.

### 2.3 Pathological examination and histopathological grading criteria

After fixation in 10% buffered formalin for 24 h, biopsy specimens underwent routine preparation on hematoxylin and eosin (HE) stained slides (16). ESD/EMR specimens were removed and photographed *in vitro* using an entity microscope. After staining with gentian violet, MLR and the surrounding mucosa were re-examined using the entity microscope. Subsequently, routine HE-stained slides were prepared according to the standardized processing protocol for gastric ESD specimens. All specimen sections were then digitized using a digital pathology section scanner. Two blinded senior pathologists independently reviewed the pathological slides while blinded to the endoscopic findings and the regional sources of the specimens, including areas R, T, and B. Only concordant diagnoses were accepted. Disagreements were resolved through further review and discussion to reach a consensus diagnosis.

Based on the updated Sydney System of Gastritis standards (17), the degree of atrophy, GIM, inflammation, and inflammatory activity were assessed in the three areas. The degree of dysplasia was evaluated according to the WHO classification of digestive system tumors (18). Each group was further classified according to the gastric intestinal metaplasia (GIM) classification based on HE staining (19–21). Type I GIM (predominantly complete GIM) referred to complete GIM or mixed GIM with complete GIM exceeding incomplete GIM. Type II GIM (predominantly incomplete GIM) referred to incomplete GIM or mixed GIM with incomplete GIM exceeding complete GIM (19). GIM typing and subtyping analyses were performed only in specimens with histologically identifiable GIM. Therefore, specimens without GIM were excluded from the denominator for these analyses. The assessment of atrophy, GIM, dysplasia, and GIM subtypes was performed under the same blinded conditions.

Motic browsing software (2.0) was used to measure and compare histopathological morphological characteristics of MLR and the surrounding background mucosa, including crypt opening (CO) width, gland length, width of intervening parts (IPs), MV alterations, eosinophilic cytoplasmic changes, jagged changes in the luminal edge of intestinal epithelium, papillary structure, and expansion and hyperplasia of foveal epithelial cells. Fields of view having equal lengths were selected from each area under a light microscope at 100 × magnification and analyzed using Image J 1.52 software to calculate the percentage of MV area (S_1_) to the area of the selected zone (S_0_). MV density was calculated as S_1_/S_0_ × 100%.

### 2.4 Statistical analysis

The Shapiro–Wilk test was used to assess the normality of continuous variables. Normally distributed continuous variables were expressed as means *±* standard deviation ( $\bar{x}\;$ *± s*), while non-normally distributed continuous variables were expressed as medians (interquartile range). Categorical variables were expressed as absolute frequencies and percentages.

For comparisons among the three predefined areas R, T, and B, one-way analysis of variance was used for normally distributed continuous variables with homogeneity of variance, and the Kruskal-Wallis H test was used for ordinal variables or non-normally distributed continuous variables. Pearson’s χ^2^ test and Fisher’s exact test were used for categorical variables, as appropriate. When the overall comparison was statistically significant, pairwise *post hoc* comparisons among areas R, T, and B were performed. Bonferroni correction was applied to adjust for multiple comparisons. All pairwise comparisons among areas R, T, and B were predefined. Two-sided *p* values < 0.05 were considered statistically significant. All statistical analyses were performed using SPSS 26.0 (IBM Corp., Armonk, NY, USA).

## 3 Results

The study finally included 56 patients diagnosed with MLR during electronic gastroduodenoscopy.

### 3.1 Endoscopic features of areas R, T, and B under image-enhanced endoscopy

**Figure 1:**
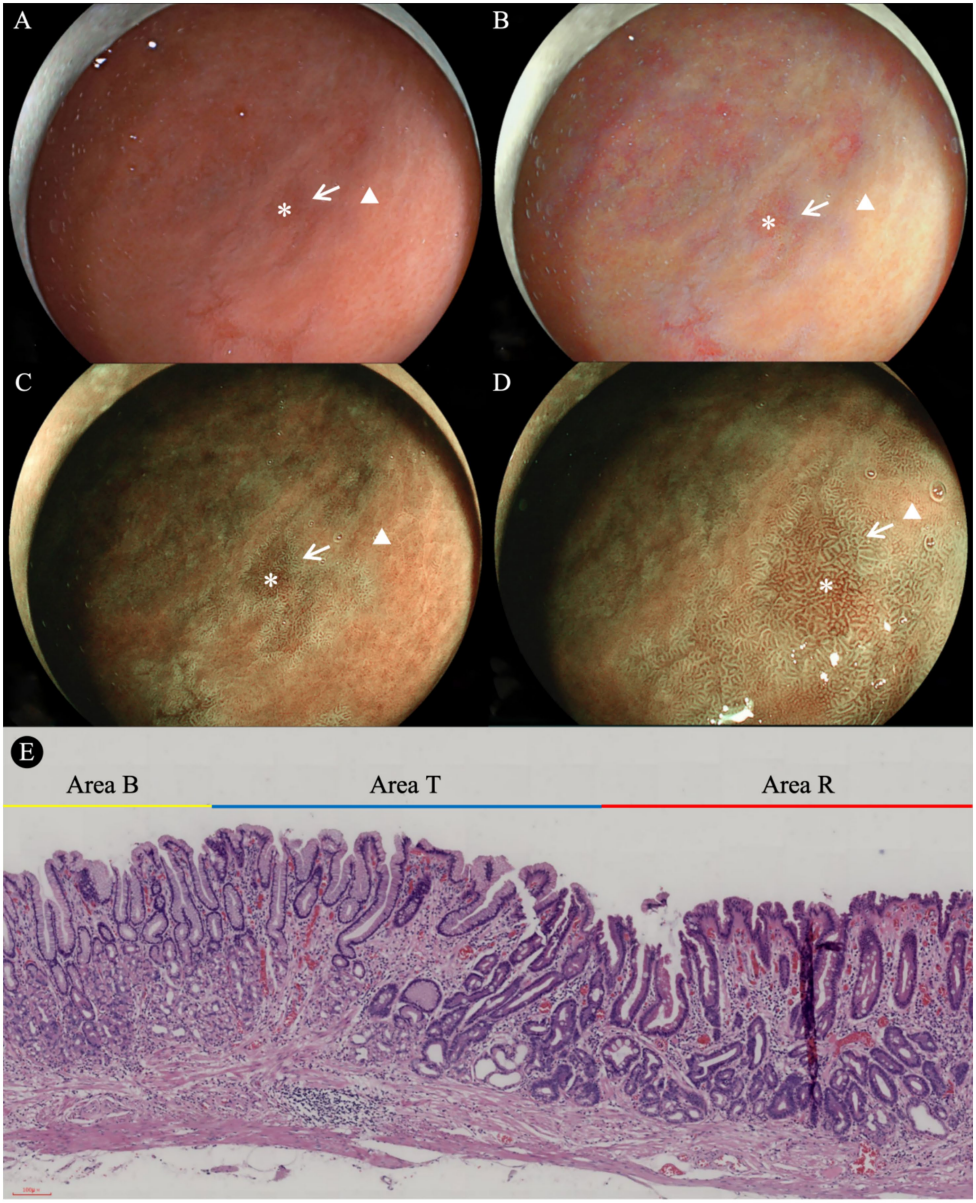
Representative endoscopic and histological findings of areas R, T, and B in the lesser curvature of the corpus. **(A)** WLI; **(B)** LCI; **(C)** BLI; **(D)** M-BLI; **(E)** Corresponding HE staining (100×). The white asterisk, white arrow, and white triangle indicate areas R, T, and B, respectively.

**Figure 2:**
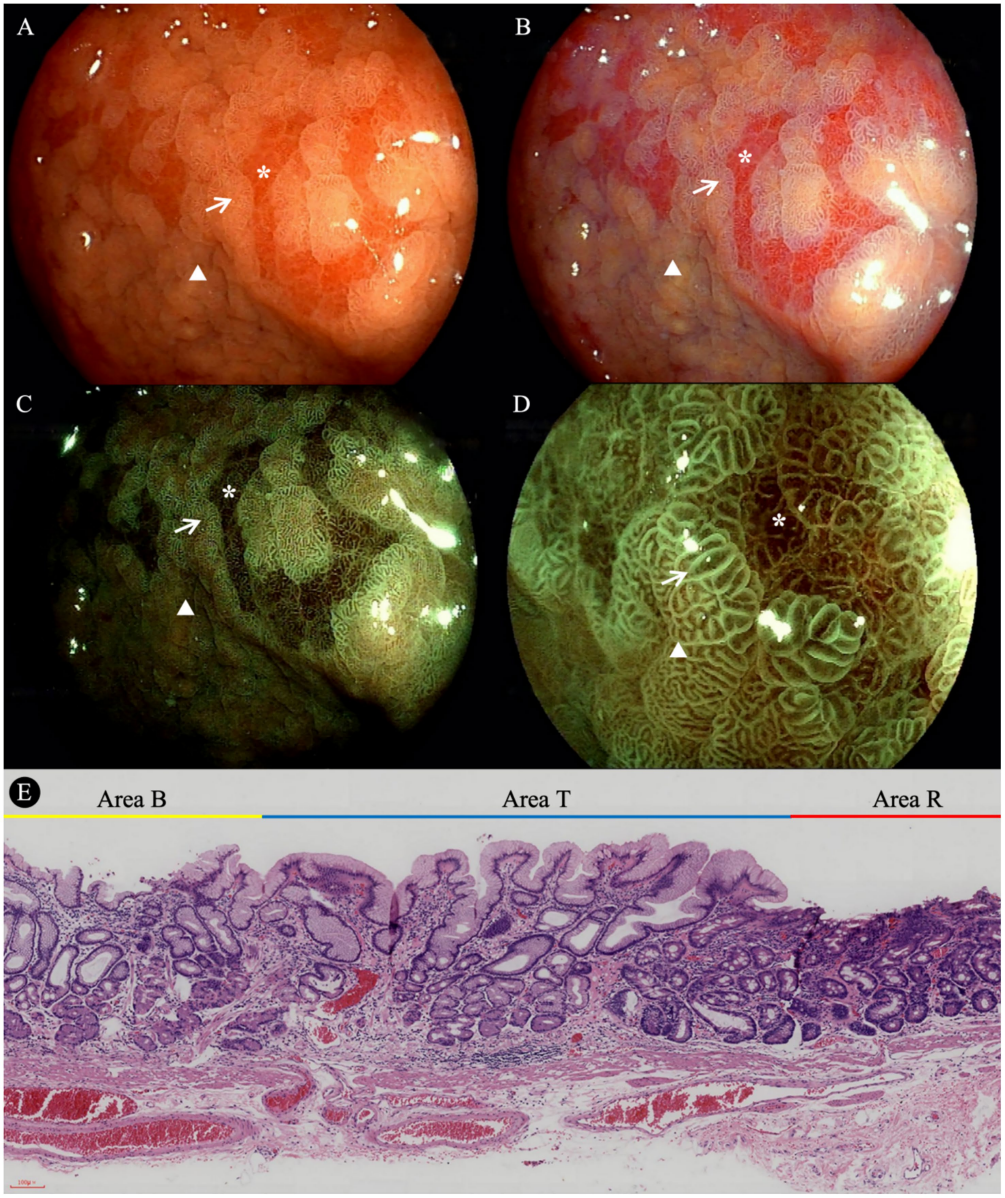
Representative endoscopic and histological findings of areas R, T, and B in the lesser curvature of the antrum. **(A)** WLI; **(B)** LCI; **(C)** BLI; **(D)** M-BLI; **(E)** Corresponding HE staining (100×). The white asterisk, white arrow, and white triangle indicate areas R, T, and B, respectively.

Gastric MLR manifested as multiple flat or depressed erythematous lesions with a consistent reddish color under WLI. It was frequently surrounded by a circle of slightly rough microprotrusions with a color slightly lighter than the surrounding mucosa. The background gastric mucosa appeared smooth with an orange-red or red and white color (Figures 1A, 2A). Under LCI, area R appeared dark red with lavender patches (purple in mist). Area T was a ring of white microprotrusions surrounding the area R, often accompanied by faint purple in mist, while area B exhibited a more uniform apricot yellow hue (Figures 1B, 2B). Under M-BLI, area R exhibited a tea-brown or dark brown color with narrow IPs, regular arrangement, and uniform size. Dilated, sometimes tortuous MVs could be observed, but the arrangement remained consistently regular. The MS pattern of area R in the body and angle of stomach showed a circular white zone surrounding round or oval COs, with a predominance of mesh-pattern MVs (Figure 1D). Tubular or villous MS patterns and coiled MV patterns predominated in the antrum (Figure 2D). Light blue crests (LBCs) were also seen in some regions of area R. Microstructures similar to those of the gastric antrum could be seen in some reddish areas of the gastric body and angle.

Area T appeared light cyan, with a cyan tubular or villous MS pattern and coiled MV pattern in both the gastric body and antrum. The IPs of the glands were widened with a regular arrangement and uniform size. Surface microstructure was not fused and the gastric groove was absent. Slightly thickened MV patterns were uniformly distributed in the glandular marginal crypt epithelium (Figures 1C,D, 2C,D).

Area B was brown, with MS patterns of the body and angle of stomach predominantly showing circular white zones around round or oval COs and mesh-patterned MVs (Figure 1D). Tubular or villous MS patterns and coiled MV patterns were prevalent in the antrum, with some portion of area B of the gastric body and angle exhibiting a microstructure similar to gastric antrum (Figure 2D).

### 3.2 Histopathology of areas R, T, and B

Of the 56 patients, 11 underwent ESD or EMR to obtain large complete specimens of MLR and the surrounding mucosa, while 45 patients underwent precise biopsies of the R, T, and B areas. The histological analysis showed mild chronic inflammation or mild atrophy in area B, while areas R and T showed gastric mucosal atrophy and GIM (Figures 1E, 2E). The updated Sydney system grading, dysplasia, GIM subtyping based on HE staining, and the histological morphologies were compared among these areas. The results of the comparisons are discussed below.

#### 3.2.1 Histological grading of areas R, T, and B using the updated Sydney system

The results for gastritis grading of areas R, T, and B using the updated Sydney system are presented in Table 1. The degrees of atrophy and GIM for area R were higher than those for areas T and B. Meanwhile, the degrees of atrophy and GIM for area T were higher than those for area B (*p* < 0.001). There were no statistically significant differences in the degree of chronic inflammation and active inflammation among the three areas.

**Table 1:** Histological grading of areas R, T, and B using the updated Sydney system.

Histological parameter	Area	Normal	Mild	Moderate	Severe	Kruskal-Wallis *H* test	
						*H-*value	*p-*value
Degree of atrophy^a^	R	0	3	21	32	117.881	< 0.001
	T	1	38	17	0		
	B	30	25	1	0		
Degree of GIM^b^	R	0	3	20	33	125.850	< 0.001
	T	10	29	17	0		
	B	50	5	1	0		
Degree of chronic inflammation	R	0	49	7	0	3.433	0.180
	T	0	49	7	0		
	B	0	54	2	0		
Neutrophil polymorph activity	R	54	2	0	0	< 0.001	1.000
	T	54	2	0	0		
	B	54	2	0	0		

Values are presented as *n*. ^a^Area R vs. area T, adjusted *p* < 0.001; area R vs. area B, adjusted *p* < 0.001; area T vs. area B, adjusted *p* < 0.001. ^b^Area R vs. area T, adjusted *p* < 0.001; area R vs. area B, adjusted *p* < 0.001; area T vs. area B, adjusted *p* < 0.001.

#### 3.2.2 Dysplasia in areas R, T, and B

Dysplasia was detected only in area R. The dysplasia rate was 19.64% in area R and 0% in areas T and B. The difference was statistically significant (*χ*^2^ = 20.560, *p* < 0.001). The rate of dysplasia for area R was higher than that for areas T and B (adjusted *p* < 0.05). Among the 11 cases of dysplasia in area R, 10 involved low-grade dysplasia (*n* = 10/56, 21.28%), while one case involved high-grade dysplasia (*n* = 1/56, 2.13%) (Table 2).

**Table 2:** GIM typing in areas R, T, and B.

Area	Total	Positive for dysplasia, *n* (%)	Negative for dysplasia, *n* (%)	Pearson’s χ^2^ test/Fisher’s exact test	
				*χ*^2^ value	*P* value
R	56	11(19.64)^a^	45(80.36)	20.560	< 0.001
T	56	0(0.00)^b^	56(100.00)		
B	56	0(0.00)	56(100.00)		

Values are presented as n (%). ^a^Area R vs. area T, adjusted *p* < 0.05; area R vs. area B, adjusted *p* < 0.05. ^b^Area T vs. area B, adjusted *p* > 0.05.

#### 3.2.3 GIM typing and subtyping in areas R, T, and B

For GIM typing and subtyping analyses, only specimens with identifiable GIM were included. GIM was identified in 56 specimens from area R, 46 specimens from area T, and 6 specimens from area B, therefore, these values were used as the denominators in Tables 3, 4.

**Table 3:** GIM typing of MLR and the surrounding gastric mucosa.

Group	Total	Complete GIM, *n* (%)	Mixed: complete exceeding incomplete GIM, *n* (%)	Mixed: incomplete exceeding complete GIM, *n* (%)	Incomplete GIM, *n* (%)	Kruskal-Wallis *H* test	
						*H-*value	*P-v*alue
R^a^	56	0 (0.0)	10 (17.85)	31 (55.36)	15 (26.79)	54.307	0.000
T^b^	46	16 (34.78)	24 (52.18)	6 (13.04)	0 (0.00)		
B	6	2 (33.33)	3 (50.0)	1 (16.67)	0 (0.00)		

^a^Area R vs. area T, adjusted *p* < 0.001; area R vs. area B, adjusted *p* = 0.004. ^b^Area T vs. area B, adjusted *p* = 1.000.

**Table 4:** GIM subtyping in areas R, T, and B.

Area	Total	Type-I GIM, *n* (%)	Type-II GIM^a^, *n* (%)	Pearson’s χ^2^ test/Fisher’s exact test	
				*χ*^2^ value	*P-*value
R	56	10 (17.86)	46 (82.14)	54.398	< 0.001
T	46	40 (86.96)	6 (13.04)		
B	6	5 (83.33)	1 (16.67)		

^a^Area R vs. area T, adjusted *p* < 0.05; area R vs. area B, adjusted *p* < 0.05; area T vs. area B, adjusted *p* > 0.05.

##### 3.2.3.1 GIM typing

The results of GIM typing in areas R, T, and B, showing statistically significant differences (*H* = 54.307, *p* < 0.001), are presented in Table 3. On further pairwise comparisons, GIM typing of area R showed statistically significant differences with that of areas T and B (adjusted *p* < 0.05). However, there was no statistically significant difference between areas T and B (adjusted *p* = 1.000). Pairwise comparisons were performed after Bonferroni correction and are shown in the table footnotes.

##### 3.2.3.2 GIM subtyping

Among specimens with identifiable GIM, type-II GIM predominated in area R, whereas type-I GIM predominated in areas T and B. The rates of type-I and type-II GIM in areas R, T, and B were 17.86% vs. 82.14, 86.96% vs. 13.04, and 83.33% vs. 16.67%, respectively, exhibiting statistically significant differences among groups (χ^2^ = 54.398, *p* < 0.001). Type-II GIM predominated in area R, while type-I GIM predominated in areas T and B. Pairwise comparisons were performed after Bonferroni correction and are shown in the table footnotes. (Table 4).

#### 3.2.4 Additional histopathological morphological changes in areas R, T, and B

The sizes of COs for areas R, T, and B, measured on scanned HE-stained slides, were 130.61 μm (112.29 μm, 147.58 μm), 70.34 μm (41.98 μm, 85.51 μm), and 38.92 μm (30.21 μm, 49.99 μm), respectively, showing statistically significant differences (*H* = 99.896, *p* < 0.001). The COs in area R were significantly larger than those in areas T and B (adjusted *p* < 0.001). Additionally, the CO size was significantly different between areas T and B (adjusted *p* < 0.001). The lengths of gastric mucosal glandular ducts for areas R, T, and B were 143.60 μm (106.06 μm, 193.44 μm), 214.16 μm (140.60 μm, 277.93 μm), and 133.97 μm (127.71 μm, 164.88 μm), respectively, exhibiting statistically significant differences (*H* = 29.805, *p* < 0.001). The glandular duct length in area T was significantly greater than that in areas R and B (adjusted *p* < 0.001), with no significant difference between areas R and B (adjusted *p* = 1.000). Furthermore, the widths of IPs for areas R, T, and B were 92.36 μm (84.13 μm, 103.02 μm), 224.51 μm (188.74 μm, 275.80 μm), and 122.14 μm (102.67 μm, 148.12 μm), respectively, with statistically significant differences among groups (*H* = 111.29, *p* < 0.001). The IP width for area T was significantly greater than that for areas R and B (adjusted *p* < 0.001), while that for area B was significantly greater than that for area R (adjusted *p* < 0.001) (Table 5).

**Table 5:** Histomorphometric measurements and microvessel density in areas R, T, and B.

Area	R median (IQR)	T median (IQR)	B median (IQR)	Kruskal-Wallis *H* test	
				*H*-value	*p-*value
CO width^a^ (μm)	130.61 (112.29, 147.58)	70.34 (41.98, 85.51)	38.92 (30.21, 49.99)	99.896	< 0.001
Gland length^b^ (μm)	143.60 (106.06, 193.44)	214.16 (140.60, 277.93)	133.97 (127.71, 164.88)	29.805	< 0.001
IP width^c^ (μm)	92.36 (84.13, 103.02)	224.51 (188.74, 275.80)	122.14 (102.67, 148.12)	111.290	< 0.001
Microvessel density^d^ (%)	3.93 (3.21, 4.17)	2.03 (1.66, 2.38)	1.47 (1.03, 2.25)	93.617	< 0.001

Data are presented as median (interquartile range), P50 (P25, P75). ^a^For CO width, area R vs. area T, adjusted *p* < 0.001; area R vs. area B, adjusted *p* < 0.001; area T vs. area B, adjusted *p* < 0.001. ^b^For gland length, area R vs. area T, adjusted *p* < 0.001; area R vs. area B, adjusted *p* = 1.000; area T vs. area B, adjusted *p* < 0.001. ^c^For IP width, area R vs. area T, adjusted *p* < 0.001; area R vs. area B, adjusted *p* < 0.001; area T vs. area B, adjusted *p* < 0.001. ^d^For microvessel density, area R vs. area T, adjusted *p* < 0.001; area R vs. area B, adjusted *p* < 0.001; area T vs. area B, adjusted *p* = 0.051. CO, crypt opening; IP, intervening part.

Image J (version 1.52) was used to measure MV density in the three groups, with values of 3.93% (3.21%, 4.17%), 2.03% (1.66%, 2.38%), and 1.47% (1.03%, 2.25%) for areas R, T, and B, respectively. The MV density for area R was significantly higher than that for areas T and B (adjusted *p* < 0.001). Similarly, the MV density for area T was higher than that for area B, but the difference was not statistically significant (adjusted *p* = 0.051) (Table 5).

**Figure 3:**
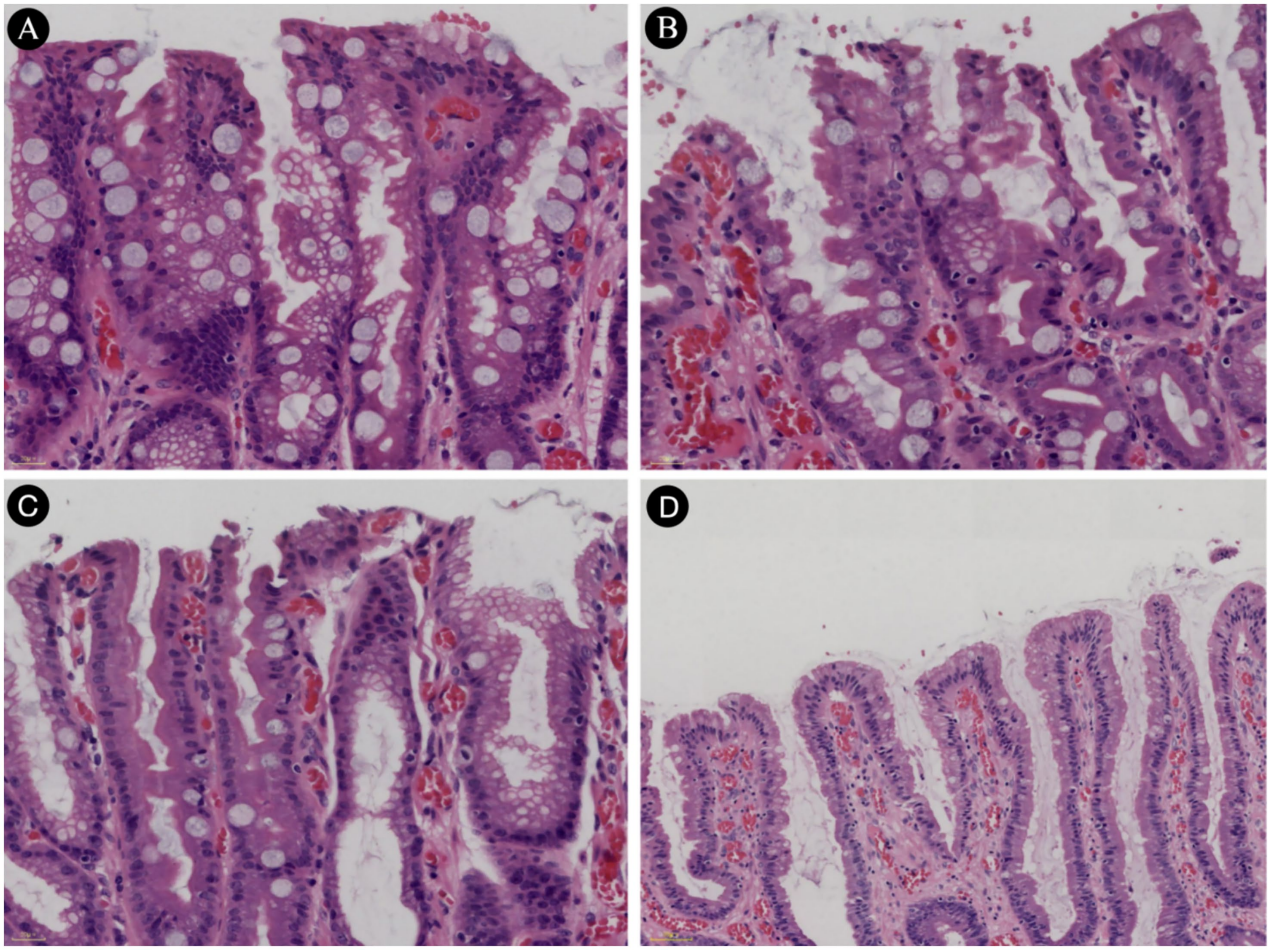
Morphological changes in areas R, T, and B. **(A)** Microvesicular changes in the cytoplasm of intestinal metaplastic epithelial cells (400×); **(B)** Jagged changes in the GIM cavity edge (400×); **(C)** Eosinophilic changes in the cytoplasm of intestinal metaplastic epithelial cells (400×); **(D)** Papillary changes in the surface mucosa (200×).

The rates of microvesicular changes in the cytoplasm, jagged changes in the luminal edge of intestinal epithelium, and papillary structure in area R were significantly higher compared to areas T and B (adjusted *p* < 0.05) (Figure 3 and Table 6). The rates of eosinophilic changes in the cytoplasm in area R were significantly higher compared to area B (adjusted *p* < 0.05). There was a statistically significant difference in the rate of papillary changes in the surface mucosa between areas T and B (adjusted *p* < 0.05), but there were no significant differences in the rates of jagged changes in the luminal edge of intestinal epithelium, eosinophilic changes, and microvesicular changes in the cytoplasm of intestinal epithelium (adjusted *p* > 0.05). There were no cases of epithelial hyperplasia in area R, while there were 46 cases (82.14%) and 2 cases (3.57%) in areas T and B, respectively. The rate of foveal epithelial cell proliferation in area T showed statistically significant differences compared to areas R and B (adjusted *p* < 0.05), but there was no statistically significant difference between areas R and B (adjusted *p* > 0.05).

**Table 6:** Additional histopathological morphological findings in areas R, T, and B.

Histopathologicalfeature	Area	Total	Positive, *n* (%)	Negative, *n* (%)	Pearson’s χ^2^ test/Fisher’s exact test	
					*χ*^2^ value	*P-*value
Jagged changes in the luminal edge of intestinal epithelium^a^	R	56	38 (67.86)	18 (32.14)	31.664	< 0.001
	T	46	8 (17.39)	38 (82.61)		
	B	6	0 (0.00)	6 (100.00)		
Eosinophilic changes in the cytoplasm of intestinal epithelium^b^	R	56	56 (100.00)	0 (0.00)	7.071	0.023
	T	46	42 (91.30)	4 (8.70)		
	B	6	5 (83.33)	1 (16.67)		
Microvesicular changes in the cytoplasm of intestinal epithelium^c^	R	56	53 (94.64)	3 (5.36)	42.239	< 0.001
	T	46	18 (39.13)	28 (60.87)		
	B	6	2 (33.33)	4 (66.67)		
Papillary changes in the surface mucosa^d^	R	56	25 (44.64)	31 (55.36)	34.286	< 0.001
	T	56	10 (17.86)	46 (82.14)		
	B	56	0 (0.00)	56 (100.00)		
Foveal epithelial cell expansion and hyperplasia^e^	R	56	0 (0.00)	56 (100.00)	118.300	< 0.001
	T	56	46 (82.14)	10 (17.86)		
	B	56	2 (3.57)	54 (96.43)		

Values are presented as *n* (%). For intestinal epithelial changes, only specimens with identifiable GIM were included in the analysis. ^a^For jagged changes in the luminal edge of intestinal epithelium, area R vs. area T, adjusted *P* < 0.05; area R vs. area B, adjusted *P* < 0.05; area T vs. area B, adjusted *P* > 0.05. ^b^For eosinophilic changes in the cytoplasm of intestinal epithelium, area R vs. area B, adjusted *p* < 0.05. ^c^For microvesicular changes in the cytoplasm of intestinal epithelium, area R vs. area T, adjusted *p* < 0.05; area R vs. area B, adjusted *p* < 0.05; area T vs. area B, adjusted *p* > 0.05. ^d^For papillary changes in the surface mucosa, area R vs. area T, adjusted *p* < 0.05; area R vs. area B, adjusted *p* < 0.05; area T vs. area B, adjusted *p* < 0.05. ^e^For foveal epithelial cell expansion and hyperplasia, area T vs. area R, adjusted *p* < 0.05; area T vs. area B, adjusted *p* < 0.05; area R vs. area B, adjusted *p* > 0.05.

## 4 Discussion

In this prospective study, we characterized the endoscopic and histopathological features of gastric MLR after successful *H. pylori* eradication. The main finding was that the central reddish area (area R) showed more advanced atrophy and GIM, a predominance of incomplete GIM, higher microvessel density, and dysplasia in some cases, compared with the surrounding transition zone (area T) and background mucosa (area B). These findings support a close correlation between the endoscopic appearance of MLR and localized high-risk mucosal. Nagata et al. (12) proposed that the redness of MLR may be due to high-density MVs surrounding the metaplastic glands. Our finding of increased microvessel density in area R is consistent with this proposed histopathological basis. Endoscopically, area R appeared dark red under LCI and showed narrow intervening parts and dilated microvessels under M-BLI. Histologically, these findings corresponded to increased microvessel density and more severe GIM. Area T appeared as a slightly pale microprotruded zone surrounding area R. Histologically, this area showed foveolar epithelial expansion and wider intervening parts, which may explain its protruded endoscopic appearance.

Since the frequency of complete and incomplete GIM varies depending on the site of the stomach, we further compared the classification of GIM of three regions in the gastric body and antrum. Among the 56 samples, 32 samples were taken from the gastric body and angle, in which the rates of type-I and type-II GIM in areas R, T, and B were 21.90% vs. 78.10, 96.20% vs. 3.80, 100.00% vs. 0.00%, respectively. There were 24 samples taken from the antrum, in which the rates of type-I and type-II GIM in areas R, T, and B were 12.50% vs. 87.50, 76.2% vs. 23.80, 75.00% vs. 25.00%, respectively. It was shown to be consistent with the results of GIM subtyping in the result.

The widened crypt openings and altered microsurface pattern in area R may correspond to expansion and remodeling of intestinal metaplastic glands. In contrast, the wider intervening parts in area T may reflect foveolar epithelial expansion and hyperplasia. These structural changes provide a plausible histological basis for the distinct M-BLI patterns observed in the different areas of MLR.

The present study demonstrated marked histological differences among areas R, T, and B, particularly the predominance of severe atrophy, incomplete GIM, and dysplasia in area R. In contrast, previous cohort studies primarily reported an association between MLR and post-eradication gastric cancer risk. Therefore, our findings should be interpreted as histopathological evidence of high-risk mucosal changes within MLR rather than direct evidence that MLR predicts future gastric cancer development. This distinction is important when interpreting previous reports suggesting that MLR is associated with gastric cancer after *H. pylori* eradication. Nagata et al. (12) identified MPE, synonymous with MLR, as a specific indicator of post-eradication gastritis. The histopathological basis of MPE was found to be GIM of the gastric mucosa, having a sensitivity of 72.7% and a specificity of 84.1%. Their study also suggested that the severity of GIM in MPE was significantly higher than that in surrounding tissues, suggesting MPE as a potential indicator for predicting post-eradication gastric cancer. Majima et al. (9) reported a significantly higher incidence of moderate-to-severe atrophy and MLR in the gastric cancer group compared to the non-gastric cancer group under LCI. Their findings supported MLR as an independent risk factor for post-eradication gastric cancer. In our study, the pathological characteristics of MLR mainly included moderate-to-severe atrophy and GIM, with a higher degree compared to the surrounding gastric mucosa, consistent with the Nagata et al. study (12). Additionally, GIM in MLR mainly consisted of incomplete GIM. Previous studies have demonstrated a significant association between incomplete GIM and gastric cancer risk (22–25). Studies have reported that the relative risk of gastric cancer in incomplete GIM patients ranges from 4 to 11 times greater than that for complete GIM patients (22–24, 26–28).

In addition to GIM type, gastric dysplasia also serves as a precursor lesion associated with gastric cancer. A national cohort study from the Netherlands (29) reported that the annual incidence rates for gastric cancer associated with atrophic gastritis, GIM, low-grade dysplasia, and high-grade dysplasia were 0.1, 0.25, 0.6, and 6%, respectively. Similarly, a cohort study (30) conducted at Ruijin Hospital revealed that the rates of low-grade dysplasia progression to high-grade dysplasia, early gastric cancer, and advanced gastric cancer over an average follow-up of 13.86 ± 32.82 months were 2.76, 6.20, and 2.76%, respectively. In our study, some MLR cases were pathologically detected as dysplasia (*n* = 11/56, 19.64%), with detection rates for low- and high-grade dysplasia being 17.86% (*n* = 10/56) and 1.79% (*n* = 1/56), respectively. Considering the relationship between MLR and gastric cancer after *H. pylori* eradication, this may be attributed to the high rate of incomplete GIM (type II GIM) and a certain proportion of dysplasia.

In this study, a portion of MLR and its surrounding gastric mucosa was examined through ESD, facilitating an accurate, continuous, and comprehensive analysis of the histopathological characteristics. At the same time, we used WLI and magnifying endoscopy for *in vivo* lesion observation, while an *in vitro* solid microscope was used for gross structure observation following resection. Point-to-point correspondence, combined with pathological ESD specimens, allowed an assessment of the relationship between endoscopic and pathological manifestations.

In clinical practice, several conditions may produce reddish or mottled gastric mucosal appearances that mimic MLR. These include reactive chemical gastropathy related to NSAID use or bile reflux, gastric antral vascular ectasia, focally enhanced gastritis associated with Crohn’s disease, autoimmune gastritis with early fundic atrophy, and portal hypertensive gastropathy. Therefore, MLR should be interpreted in the context of *H. pylori* eradication status, lesion morphology, distribution, image-enhanced endoscopic findings, and histopathological correlation.

It is important to acknowledge the limitations of our study. First, this was a single-center study with a relatively small sample size, and its results require validation through larger-scale, multicenter prospective studies. Second, formal independent double endoscopic reading was not performed for all cases, which may have introduced observer bias in the diagnosis and interpretation of MLR. Third, the study did not include a non-MLR control group or longitudinal follow-up data, therefore the relationship between MLR and future gastric cancer development could not be directly assessed. Fourth, potential confounding factors, including medication history, bile reflux, and lifestyle factors, were not fully controlled. Finally, the subjective nature of evaluating endoscopic signs and updated Sydney pathological scores may have introduced an observer bias. To reduce bias, all assessments were performed by endoscopists, having an experience of more than 1,000 endoscopic procedures, who underwent unified LCI training. To reduce histological assessment bias, pathological evaluations were independently performed by two senior pathologists who were blinded to the endoscopic findings and the sampling areas (areas R, T, and B), and discrepancies were resolved by consensus. Additionally, the updated Sydney system scores and intestinal classification, having a high consistency, were used to reduce the risk of bias (19, 31). However, standardized diagnostic criteria for MLR, including a predefined minimum surface area, remain to be established in future studies.

In conclusion, the central reddish area of gastric MLR under image-enhanced endoscopy usually presented as a circular white zone surrounding round or oval COs with a mesh MV pattern. Tubular or villous MS patterns and coiled MV patterns predominated in the antrum. A ring of light microbulges surrounding the reddish area often manifested as a large tubular or villous MS pattern with coiled MV pattern. The histological features of MLR included moderate-to-severe atrophy and GIM, incomplete GIM, increased MV density in the red area, and widening of the transition zone IPs. The rate of incomplete GIM and dysplasia was higher in area R. These findings may partly explain why previous studies have associated MLR with an increased risk of gastric cancer after *H. pylori* eradication. Therefore, MLR, particularly area R, may serve as an endoscopic marker of localized high-risk gastric mucosal changes after *H. pylori* eradication. Further multicenter studies with control groups and longitudinal follow-up are needed to clarify whether these findings translate into an increased future risk of gastric cancer.

## Data Availability

Due to the sensitive nature of the data, including patient information, access is restricted. Data are available from the corresponding author upon reasonable request and with permission from the Biomedical Ethics Committee of the 900th Hospital of PLA Joint Logistic Support Force.
